# Analyzing the Impact of Greenhouse Planting Strategy and Plant Architecture on Tomato Plant Physiology and Estimated Dry Matter

**DOI:** 10.3389/fpls.2022.828252

**Published:** 2022-02-15

**Authors:** Yue Zhang, Michael Henke, Yiming Li, Demin Xu, Anhua Liu, Xingan Liu, Tianlai Li

**Affiliations:** ^1^College of Horticulture, Shenyang Agricultural University, Shenyang, China; ^2^Key Laboratory of Protected Horticulture, Ministry of Education, Shenyang, China; ^3^National & Local Joint Engineering Research Center of Northern Horticultural Facilities Design & Application Technology (Liaoning), Shenyang, China; ^4^Plant Sciences Core Facility, CEITEC-Central European Institute of Technology, Masaryk University, Brno, Czechia; ^5^Leibniz Institute of Plant Genetics and Crop Plant Research (IPK), Stadt Seeland, Germany; ^6^College of Engineering, Shenyang Agricultural University, Shenyang, China

**Keywords:** functional-structure plant modeling (FSPM), planting strategy, plant architecture, photosynthesis, partial least squares path modeling (PLS-PM), GroIMP

## Abstract

Determine the level of significance of planting strategy and plant architecture and how they affect plant physiology and dry matter accumulation within greenhouses is essential to actual greenhouse plant management and breeding. We thus analyzed four planting strategies (plant spacing, furrow distance, row orientation, planting pattern) and eight different plant architectural traits (internode length, leaf azimuth angle, leaf elevation angle, leaf length, leaflet curve, leaflet elevation, leaflet number/area ratio, leaflet length/width ratio) with the same plant leaf area using a formerly developed functional–structural model for a Chinese Liaoshen-solar greenhouse and tomato plant, which used to simulate the plant physiology of light interception, temperature, stomatal conductance, photosynthesis, and dry matter. Our study led to the conclusion that the planting strategies have a more significant impact overall on plant radiation, temperature, photosynthesis, and dry matter compared to plant architecture changes. According to our findings, increasing the plant spacing will have the most significant impact to increase light interception. E–W orientation has better total light interception but yet weaker light uniformity. Changes in planting patterns have limited influence on the overall canopy physiology. Increasing the plant leaflet area by leaflet N/A ratio from what we could observe for a rose the total dry matter by 6.6%, which is significantly better than all the other plant architecture traits. An ideal tomato plant architecture which combined all the above optimal architectural traits was also designed to provide guidance on phenotypic traits selection of breeding process. The combined analysis approach described herein established the causal relationship between investigated traits, which could directly apply to provide management and breeding insights on other plant species with different solar greenhouse structures.

## Introduction

**FIGURE 1:**
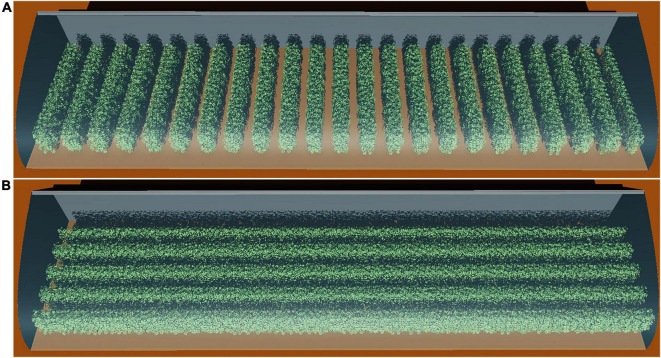
Snapshot of the 3D model of a 30 m long Liaoshen-solar greenhouse (LSG) including the tomato canopy with N–S row orientation **(A)** and E–W row orientation **(B)**. While a typical LSG is about 60 m, a 30-m version was used for simulation to reduce computation time and so enables us to simulate more scenarios. A reduction in dimensions at this low rate still allows simulations without any impact on the overall simulated results.

Chinese solar greenhouses enable growers to control the surrounding environment and make them more suitable for plant growth (Zhang S. et al., 2020), and this is widely used in agricultural industries in China (Tong et al., 2013). Greenhouse management is usually based on the empirical knowledge and experience of the farmers (Choab et al., 2019), resulting in a large variety of greenhouse management techniques and skills. However, this way only allows adaptations in small steps in different directions without considering an overall optimization goal. It therefore might lead to wrong directions or get stuck into a local optimal. The rapid technology development in recent years enables systematic *in silico* studies of different plant architectures and their interactions with their environment to simulate plant microenvironment conditions and physiology like microlight climate or photosynthesis on high resolution (Zhang Y. et al., 2020; Zhang Y. et al., 2021) under different conditions, such as planting pattern or plant architecture (Figure 1). The greenhouse structure, planting strategy, and plant architecture are interdependent to each other, and knowing dynamic dependencies between these traits can make sense, for example, to profile photosynthesis performance or to instruct the greenhouse management. Those must be considered within realistic simulations, aiming to optimize plant growth conditions and, ultimately, increasing yield (Chelle, 2005).

Planting strategies have a direct impact on the interplant microlight climate, causing shading that hampers not only optimal light but also temperature conditions within the canopy, which are directly related to photosynthesis (Campos et al., 2017) and therefore might result in usually unwanted light competitions between plants that typically led to an increased structural growth but decreased fruit growth. General planting strategies include many aspects, such as overall plant density, plant and furrow distances, row orientation, planting pattern, etc. However, it is often considered as a given, immutable law for growers because needed time and cost of investigating new configurations are far too high. The effects of planting strategies on tomato production performance depend heavily on various factors, such as the greenhouse structure, incoming radiation level defined by the latitude, and the plant architecture as well. Many studies tried to sort out these interconnected relationships for single factors but rarely investigated the combination of two or more. In olive hedgerow systems, the north–south orientation (N–S orientation) out-yielded east–west orientation (E–W orientation) in eight out of eleven cases. Other cases showed that at higher latitudes, the E–W orientation might provide better light interception, as reviewed by Trentacoste et al. (2015). Maddonni et al. (2001) studied maize canopy azimuth spatial distributions with square and rectangular patterns. A narrow-wide-row planting pattern also improves the light interception across maize leaves (Feng et al., 2019). For greenhouse tomatoes, van der Meer et al. (2021) found that N–S orientation had higher light uniformity and slightly lower overall light absorption and photosynthesis. They reached the conclusion that row orientation had minimal influence on the plant light absorption and photosynthesis in the glasshouse. On the other hand, Sarlikioti et al. (2011b) suggested that light absorption on the N–S orientation is higher than E–W orientation in both summer and wintertime at a latitude of 52° north. Ohashi et al. (2020) investigated tomato plants with different furrow distance adaptations (60–160 cm). They found that the middle layer was saturated with solar radiation at 100 cm and the lower layer at 120 cm. A suitable planting strategy can improve plant canopy structure, ventilation, light transmission performance, increase the utilization of light energy, and promote plant growth and development.

Plant architecture, starting from leaf shape and orientation, over branching pattern, or the number of tillers, has a significant impact on plant physiognomy. It is directly related to the plant light interception, microtemperature climate, and therefore, photosynthesis distribution within the plant and plant stands (Sarlikioti et al., 2011a; Rötter et al., 2015; Moin-E-Ddin Rezvani et al., 2021). Current molecular technologies put scientists and breeders in the position to select specific plant architecture phenotypic traits using gene editing or crossbreeding methods. However, there is still a lack of comprehensive theoretical guidance on which kind of phenotypic traits performs better (Ichihashi et al., 2014). Falster and Westoby (2003) indicated that average leaf size is the dominant factor determining self-shading. According to their studies, plants with shallow-angled leaves performed better on light interception and carbon gain. Zhu et al. (2015) revealed that intercropping with plasticity was 23% higher on the light capture than monocultures. Burgess et al. (2017) showed that rice with steeper leaves transmitted more light into the lower canopy layers. Tang et al. (2019) found the branching angle has a significant impact on light interception for loquat trees. Rowland et al. (2020) reported that tomato plants with round leaf shapes have an increased fruit-sugar content and significantly higher yield.

The studies mentioned above were all conducted under only one changing factor. To study the complex relationships between greenhouse structure, plant architecture, and planting strategies, taking only one of these factors into consideration is not sufficient to represent all details, which clearly shows the demand to identify a more efficient and accurate approach, which is flexible enough to, for example, change parameters for systematic scenario simulations, particularly for 3D-plant architecture and plating pattern simulations.

The functional–structural plant modeling (FSPM) approach is a well-established plant modeling approach (Vos et al., 2010) widely used to investigate all kinds of plant growth-related aspects, like transport process (Albasha et al., 2019), transpiration (Zhu et al., 2018), photosynthesis (Chen et al., 2014), growth behavior, pattern (Evers et al., 2010; Auzmendi and Hanan, 2020), etc. Specialized modeling tools, e.g., GroIMP: (Hemmerling et al., 2008; Kniemeyer, 2008), GreenLab: (Yan et al., 2004), or OpenAlea: (Pradal et al., 2008) allow accurate3D modeling and light simulations (Henke et al., 2016), which are designed to explore the complex relationship between the plant structure and its underlying physiological and ecological process that can be explained using mechanical principles (Louarn and Song, 2020). Countless studies have been carried out on the study of plant-architecture-related light interception using FSPM (Buck-Sorlin et al., 2011; Cieslak et al., 2011; Chen et al., 2014; Utama, 2015; Jung et al., 2018; Kim et al., 2020). By using the light simulation techniques based on ray-tracing like inverse Monte Carlo path tracing technology (Hemmerling et al., 2008), the complex microlight environment within 3D structures, e.g., between the canopy and greenhouse structure, was able to be simulated on high resolution (Vermeiren et al., 2020). de Visser et al. (2014) showed that using ray tracing and a detailed FSP model could compute optimal LED interlighting positions for tomato plants. Buck-Sorlin et al. (2020) determined the optimal LED position and leaf area index (LAI) for greenhouse cucumber production systems using FSPM. All these studies demonstrated the impressive capabilities of 3D light simulations within the FSPM approach.

The objective of this study was to estimate the effects of different combinations of influencing factors between tomato plant architecture and planting strategy on the leaflet level radiation interception, temperature profiles, and carbon assimilation for the widely used Chinese Liaoshen-solar greenhouse type (LSG) in China, and ultimately finding an optimal combination of plant architecture and planting strategy, which is suitable for LSG planting. A partial least-squares path modeling (PLS-PM) approach was further performed to estimate the quantitative relationships between plant architecture and planting strategies under LSG microclimate conditions.

## Materials and Methods

### Experimental Site and Model Description

**FIGURE 2:**
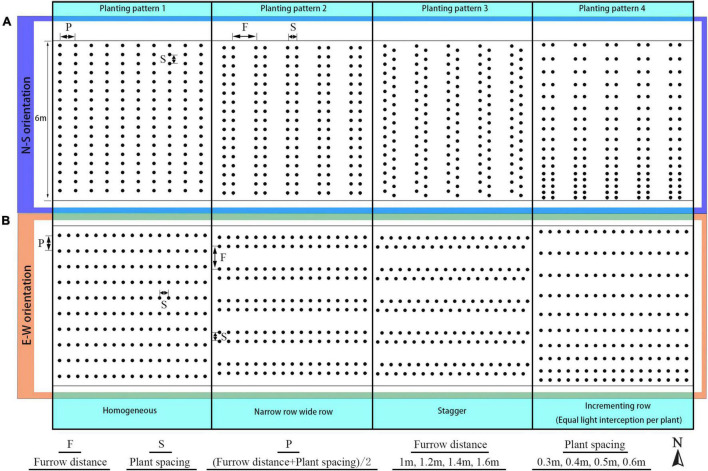
Schematic representation of the N–S row **(A)** and E–W row orientation **(B)** with four planting patterns, namely: homogeneous (planting pattern 1), narrow row wide row (planting pattern 2), stagger (planting pattern 3), and incrementing row (planting pattern 4).

The reference greenhouse structure located at Shenyang Agricultural University (41°49′N, 123°34′E) has a dimension of 60 m length, 8 m span, 4 m ridge height, and a roof projection of 1.5 m, with a 2.5 m high north wall. Tomato crops were grown inside the greenhouse with N–S orientation, same as the planting pattern 2 (Figure 2), with a furrow distance of 1 m and a plant spacing of 0.4 m. Detailed measurements for this solar greenhouse were conducted with measurements of outdoor and indoor solar radiation, temperature, wind speed, humidity, tomato canopy radiation, temperature, and photosynthesis at various canopy depths (Zhang Y. et al., 2020; Zhang Y. et al., 2021).

The three-dimensional virtual sunlight model, solar greenhouse model, and tomato model, as described in Zhang Y. et al. (2020) was constructed using the 3D open-source modeling platform GroIMP (Kniemeyer, 2008). Zhang Y. et al. (2020) predicted the organ-level temperatures of tomato leaflets inside the canopy on a sunny day, followed by simulation of the tomato canopy organ-level photosynthesis on sunny and cloudy days with the predicted datasets agreeing well with the measured data (Zhang Y. et al., 2021). In this study, we used the three-dimensional models as described above to reconstruct the tomato canopy (Supplementary Figure 1), mimicking the plant architectures and planting strategies, and then used the validated extended temperature and photosynthesis models to calculate the tomato canopy light interception, temperature, stomatal conductance, and photosynthesis performance with each case under the same greenhouse microclimate conditions. Plant-assimilated dry matter was calculated using individual plant leaflet net photosynthetic rate (gross photosynthesis minus photorespiration and dark respiration) converted to CO_2_ assimilation, and taking the total plant leaf area into consideration at the same time.

### Analysis Methods

#### Planting Strategies

The arrangement for the planting strategies investigated in this study is shown in Figure 2. First, planting patterns with the same plant density were divided into four by each orientation, resulting in a total of eight planting patterns for each density configuration; planting pattern 1 was the pattern with homogeneous row spacing which was calculated by half of the furrow distance and plant spacing added together (P). In planting pattern 2, the homogeneous row was split into a narrow row-wide row pattern, with wide row distance being furrow distance (F), narrow row distance equal to plant spacing (S). In planting pattern 3, the plants between two adjacent rows were moved into stagger style with the furrow distance and plant spacing being identical to pattern 2. As for planting pattern 4, plant spacing inside each row were changed into incrementing pattern (Eqs 1 and 2) with mean plant spacing kept the same as former patterns. Second, the furrow distance of each planting pattern configuration was increased from 1 to 1.6 m with a 0.2-m interval. Third, the plant spacing of each planting pattern was also increased from 0.3 to 0.6 m with a 0.1-m interval. To keep the plant density consistent for all eight planting patterns, two-dimension of plant special were set as the same value. It resulted in a total of 128 (8 × 4 × 4) different planting patterns investigated in this study. The simulation of canopy light, thermal, and photosynthesis conditions for each planting pattern was repeated for each time point at an interval of 30 min on the winter solstice day (22 December, day 355 of the year 2014) between 8.30 a.m. and 4.30 p.m., leading to a total of 2,048 (16 × 128) planting strategy simulations carried out.

The calculation of incrementing row was an arithmetic sequence:


(1) $$\text{W}=\text{n}\,\left(\text{P}\,S\right)+\frac{\text{n}\,\left(\text{n}-1\right)\,\text{d}}{2}$$

(2) n=W/P⁢S

where *W* is the width of the planting area (m), *n* stands for the number of a single line of plants from north to south, PS represents the plant spacing, and *d* is the common difference between every two adjacent plants.

#### Plant Architecture

In this study, we investigated the different effects of grown-up tomato crop architecture on the light interception, temperature, and photosynthesis under the same planting pattern (pattern 1 with N–S orientation, *P* = 0.65 m, *S* = 0.3 m). A static 3D tomato plant model of an adult tomato plant with 21 internodes and a maximal height of ∼1.8 m was used as the base for all following adaptations (Supplementary Figure 1A and Table 1).

**TABLE 1:** General simulation parameters for the LSG model and the reference tomato plant model.

Description	Value (range)	Unit
**Greenhouse**		
Front cover (L, W, H)	30, 8.2, 0.00015	Meter
Wall (L, W, H)	30, 2.5, 0.48	Meter
Roof (L, W, H)	30, 2.12, 0.3	Meter
Soil (L, W, H)	30, 8, 0.5	Meter
**Weather parameter of winter solstice day**		
Outdoor average radiation (12 p.m.)	435	W m^–2^
Outdoor temperature (12 p.m.)	8.50	°C
Simulated indoor temperature (12 p.m.)	24.63	°C
Indoor relative humidity	63	%
CO_2_ concentration inside greenhouse	321	ppm
**Reference plant architecture used for simulation**		
Maximal leaf rank per plant	21	–
Final height of an adult plant	1.85	Meter
Paired leaflet number per leaf rank (1–21)	7, 6, 6, 8, 7, 7, 6, 5, 6, 6, 6, 7, 7, 6, 7, 6, 6, 4, 4, 4, 6, 6	–
Average horizontal angle of petiole	55	°
Average internode length per rank	0.15, 0.20, 0.08, 0.09, 0.08, 0.07, 0.09, 0.08, 0.07, 0.07, 0.09, 0.08, 0.08, 0.07, 0.08, 0.08, 0.06, 0.08, 0.07, 0.06, 0.06, 0.06	Meter
Average leaf elevation angle	0	°
Average leaf azimuth angle	140	°
Average leaflet elevation angle	0	°
Average leaflet length per leaf rank (1–21)	0.10, 0.10, 0.10, 0.10, 0.09, 0.12, 0.14, 0.15, 0.10, 0.14, 0.12, 0.10, 0.15, 0.13, 0.14, 0.11, 0.12, 0.10, 0.10, 0.10, 0.07, 0.07	Meter
Range of internode diameter linear interpolated from bottom to top	(0.0025, 0.01)	Meter

**FIGURE 3:**
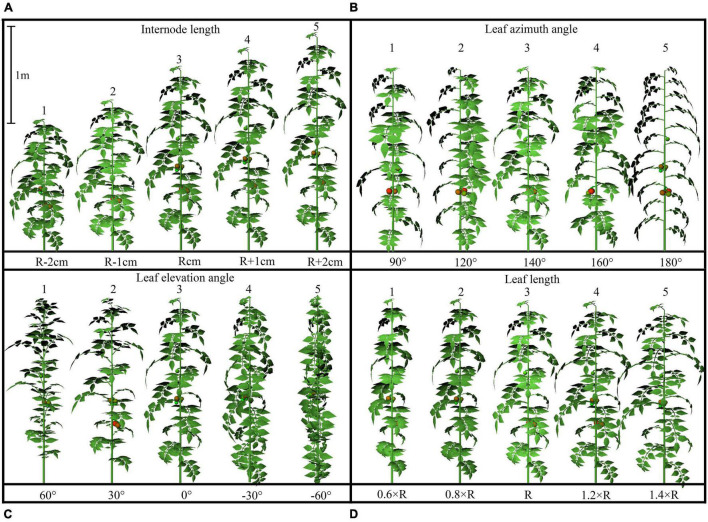
Detailed configurations of the plant architectural traits. Each plant architecture group was simulated individually with five adaptations for each scenario. Each adaptation was applied to every leaf rank of tomato plant, with visual 3D representation of each leaf level adaptation, namely: internode length (R –2 cm, R –1 cm, R cm, R + 1 cm, R +2 cm) **(A)**, leaf azimuth angle (90, 120, 140, 160, 180°) **(B)**, leaf elevation angle (60, 30, 0, –30, –60°) **(C)**, and leaf length (0.6 × R, 0.8 × R, R, 1.2 × R, 1.6 × R) **(D)**.

**FIGURE 4:**
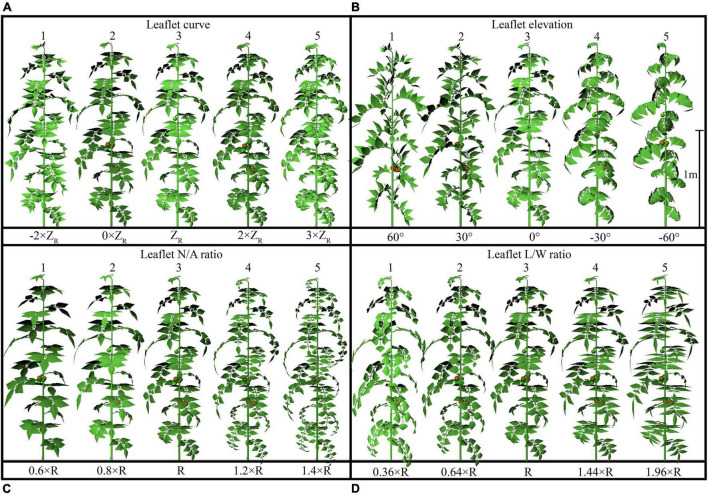
Detailed configurations of the plant architectural traits. Each plant architecture group was simulated individually with five adaptations for each scenario. Each adaptation was applied to every leaf rank of tomato plant, with visual 3D representation of each leaf-level adaptation, namely: leaflet curve (–2 × Z_*R*_, 0 × Z_*R*_, Z_*R*_, 2 × Z_*R*_, 3 × Z_*R*_) **(A)**, leaflet-elevation angle (60, 30, 0, –30, –60°) **(B)**, leaflet number/area ratio (0.6 × R, 0.8 × R, R, 1.2 × R, 1.4 × R) **(C)**, and leaflet length/width ratio (0.36 × R, 0.64 × R, R, 1.44 × R, 1.96 × R) **(D)**. Z_*R*_ being the reference Z-axis value of each leaflet modeling point. The different leaflet curvature adaptations were achieved by changing the Z-axis value of each point and then rescaled according to the reference length of the leaflet.

The configurations of plant architecture were divided into three groups [leaf-level architectural shapes (Figure 3), leaflet-level architectural shapes (Figure 4), and plant leaf area changing shapes (Supplementary Figure 2)] based on the level of alteration that has been made. (1) For the leaf arrangement, simulations were carried out to analyze the influence of different internode length (R−0.02 to R + 0.02 m), leaf length (R × 0.6 to R × 1.4), leaf elevation angle (−60 to 60°), and leaf azimuth angle (90 to 180°) (or known as the phyllotactic angle). The detailed 3D visual representation of each architectural shape is shown in Figure 3; (2) for the leaflet arrangement on each rank, four subset architectural shapes were included, with leaflet curve (−2 × Z_*R*_ to 3 × Z_*R*_), leaflet elevation angle (60 to −60°), leaflet number/area ratio (R × 0.6 to R × 1.4), and leaflet length/width ratio (R × 0.36 to R × 1.96), as shown in Figure 4. The total plant leaf area of the above eight architectural shape adaptations was kept the same as the reference structure. The different leaflet curve adaptations were achieved by changing the reference *Z*-axis value of each leaflet modeling point (Z_*R*_) and then rescaled according to the reference length of the leaflet (Supplementary Figure 3); (3) for the plant leaf area changing arrangement on each rank, four subset architectural shapes were included, with the leaflet number (R−2 to R + 2), leaflet area (R−0.02 to R + 0.02 m), leaflet length (L_*R*_ × 0.6 to L_*R*_ × 1.4), and leaflet width (W_*R*_ × 0.6 to W_*R*_ × 1.4), as shown in Supplementary Figure 2.

All the above-mentioned architectural arrangements were carried out on the whole plant level of each leaf rank, which led to 60 (12 × 5) different scenarios with time points repeated at 30-min intervals on the winter solstice day (22 December, day 355 of the year 2014) between 8.30 a.m. and 4.30 p.m., leading to 960 (16 × 60) plant architecture simulations, with other conditions kept the same. The other architecture traits were kept the same as the reference value while changing the above architecture arrangements.

### Statistical Analysis

The simulated data for all scenarios, including the planting strategies and plant architectures, were compiled into one single database (CSV-file) and further used to perform the PLS–PM in S_*MART*_PLS 3.0 (Ringle et al., 2015). The PLS–PM analysis was used to explore the cause-and-effect relationships between the simulated variables (such as field, physiological, and morphological data) through modeled latent variables. PLS–PM is well-known for determining the unknown connections between latent variables with large-scale datasets (Rowland et al., 2020). Regarding the simulation process of PLS–PM, 1,000 bootstraps were performed to obtain *R*^2^ values of the latent variable, statistical significance, and confidence intervals of the path coefficients. The path coefficients (i.e., standardized partial regression coefficients) serve as the strength and direction of the causal relationships between variables (direct effects). Indirect effects are the multiplied coefficients between the predictor variable and the response variable (all possible paths other than the direct effect) (Barberán et al., 2014). The latent variables of biological relationships were combined to determine the best path model.

## Results

### Planting Strategy

**FIGURE 5:**
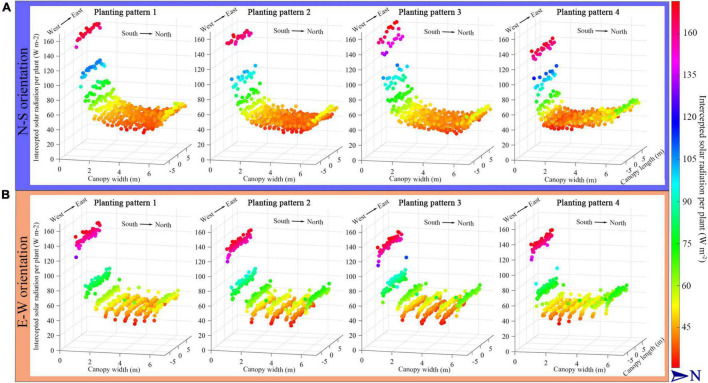
The midday solar radiation interception per plant of the four planting patterns under N–S orientation **(A)** and E–W orientation **(B)**. Each sphere, height and color represents the intercepted solar radiation intensity of a whole plant as sum of all absorbed radiation form all leaves and leaflets of a plant.

The simulated tomato canopy with four planting patterns, each under the two different orientations (N–S orientation and E–W orientation, leading to eight planting patterns), indicating the intercepted solar radiation per plant, is shown in Figure 5. To eliminate the influence of the boundary effect, the middle part of the tomato canopy was chosen to illustrate the results (−5 to 5 m to the middle of greenhouse length, with 6 m width of planting area). The simulated radiation levels at the south part of the canopy were all significantly higher than the middle and north part in eight scenarios due to the structure of the Chinese solar greenhouse south cover. The intercepted radiation levels of the four planting patterns under E–W orientation were higher than that of the N–S orientation in the middle and back part of the canopy. According to the figure, the incrementing row (planting pattern 4) of all orientations had a more significant amount of solar radiation intercepted on the back part of the canopy.

#### Plant Spacing

**FIGURE 6:**
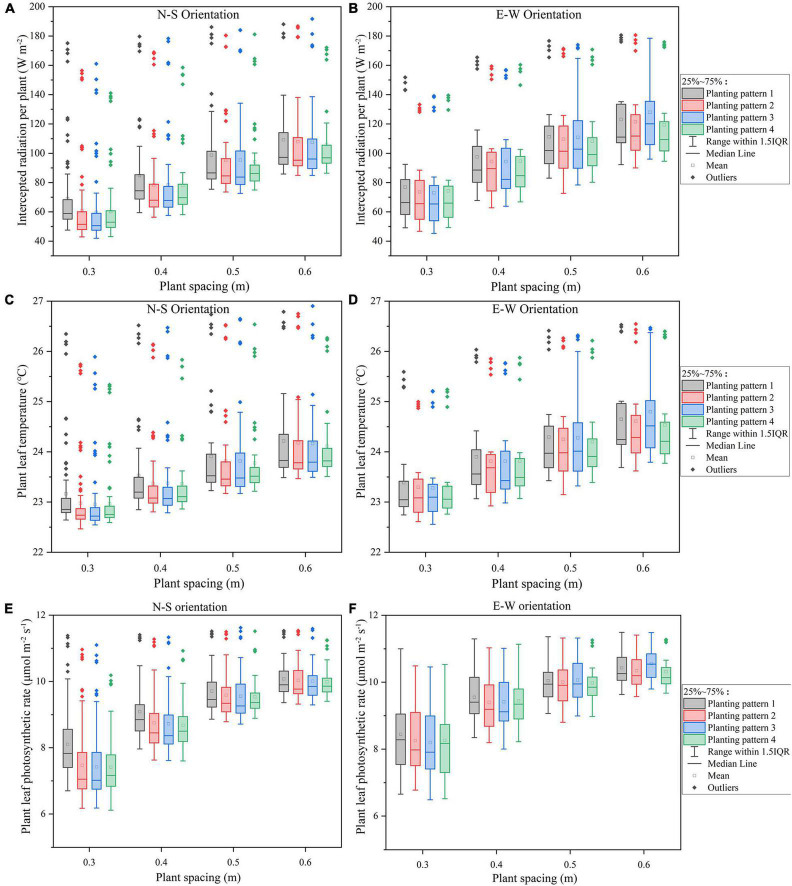
The midday plant solar radiation interception **(A,B)**, leaf temperature **(C,D)**, photosynthesis **(E,F)** of the four planting patterns under N–S orientation **(A,C,E),** and E–W orientation **(B,D,F)** as the plant spacing increases.

**FIGURE 7:**
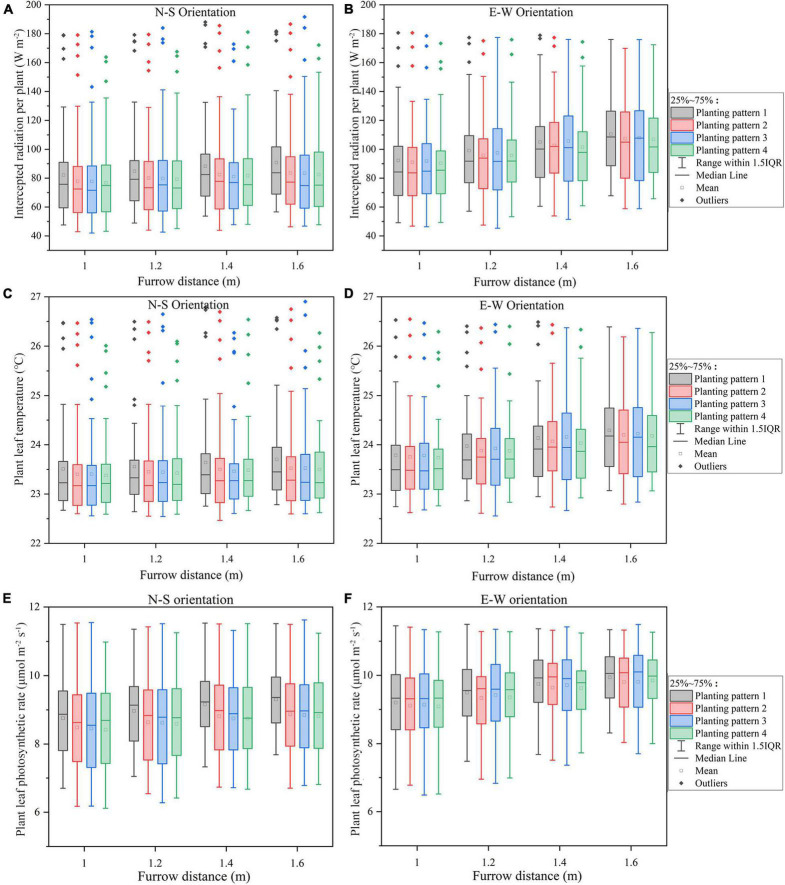
The midday plant solar radiation interception **(A,B)**, leaf temperature **(C,D)**, photosynthesis **(E,F)** of the four planting patterns under N–S orientation **(A,C,E)**, and E–W orientation **(B,D,F)** as the furrow distance increases.

The intercepted radiation (Figures 6A,B), temperature (Figures 6C,D), and photosynthesis (Figures 6E,F) of all the eight planting patterns with plant spacing ranging from 0.3 to 0.6 m were simulated to investigate the effect of plant spacing on the canopy microclimate. As shown in Figure 6, the homogeneous pattern (planting pattern 1) absorbed the most amount of radiation compared to other planting patterns in both the orientations; however, incrementing row pattern (planting pattern 4) performed the best on uniformity of plant light interception in both N–S and E–W orientation by taking into consideration the outliers. Compared to furrow distancing (Figure 7), the plant radiation, temperature, and photosynthesis increased dramatically as the plant spacing increased in both orientation scenarios (Figure 6); N–S orientation performed better on the light uniformity (Figure 6A) and E–W orientation performed better on overall light interception (Figure 6B).

#### Furrow Distance

The overall standard deviations of intercepted radiation, temperature, and net photosynthesis per plant in the furrow distance increment simulations were similar across all N–S orientations (Figures 7A,C,E), in sharp contrast to the plant spacing increment simulations (Figures 6A,C,E). Increasing the furrow distance on E–W orientation, however, will lead to an increase in the light interception. E–W orientation still performed better on overall light interception than N–S orientation. Incrementing row pattern performed the best on uniformity of plant light interception in both the N–S and E–W orientation (taking considerations of the outliers).

#### Row Orientation

**FIGURE 8:**
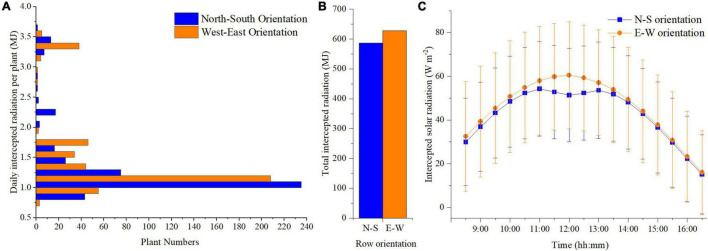
Comparison of the daily total intercepted radiation with corresponding number of plants of N–S orientation and E–W orientation of planting pattern 4 **(A)**. Comparison of single plant daily accumulated solar radiation of N–S orientation and E–W Orientation of planting pattern 4 (incrementing row) **(B)**. Comparison of plant average solar radiation intensity of N–S orientation and E–W Orientation of planting pattern 4 (incrementing row) **(C)**.

The planting pattern 4 (incrementing row) of N–S and E–W orientation with the highest plant density (*F* = 1 m, *S* = 0.3 m) was selected based on the above results to further compare the detailed difference between the two orientation scenarios. Plants located at the front, middle, and back of the canopy (point a–c, respectively) were selected to analyze the organ level difference between the two orientations (Supplementary Figure 4). More or less similar trends can be observed in the two orientation scenarios simulations (Supplementary Figure 5). However, some difference still can be found at point c, where the upper part of the plant in E–W orientation can intercept a slightly higher amount of solar radiation than N–S orientation at midday. Based on the statistical analysis of daily total intercepted radiation, the canopy in E–W orientation will intercept more solar radiation than N–S orientation during the day (Figure 8B), and the number of plants exposed to high-radiation in the E–W orientation was more significant than that of the N–S orientation (Figure 8A). This explains why the standard deviation in E–W orientation was higher than the N–S orientation during the day (Figure 8C). Due to the canopy structure of the N–S orientation, the back parts of the plants were more shaded at noon. Thus, the E–W orientation was higher than the N–S orientation with more considerable variations during midday (from 11:00 a.m. to 1:00 p.m.) (Figure 8C).

**FIGURE 9:**
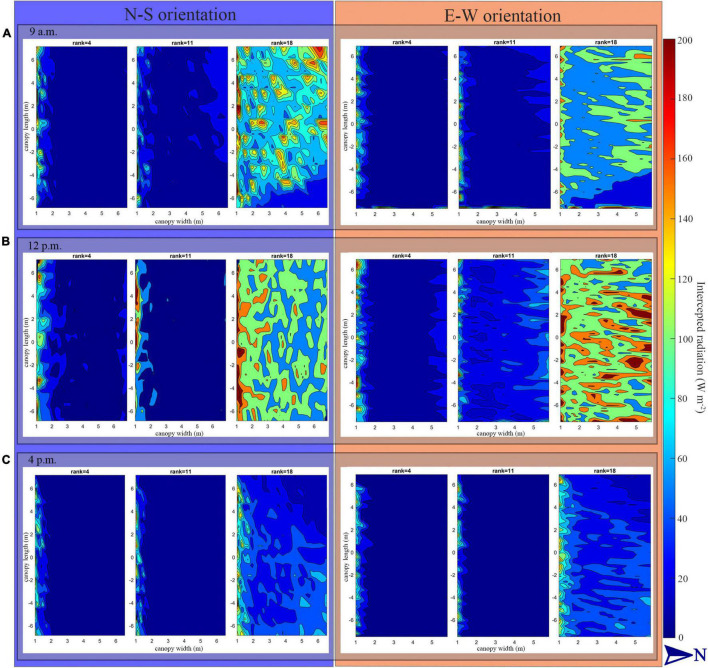
Heat map of intercepted solar radiation of tomato canopy at lower (rank = 4), middle (rank = 11) and upper layer (rank = 18) at 9:00 a.m. **(A)**, 12:00 p.m. **(B)**, and 4:00 p.m. **(C)** under N–S orientation and E–W orientation of planting pattern 4 (incrementing row).

The heatmap in Figure 9 describes the solar radiation interception of the lower (rank = 4), middle (rank = 11), and upper layer (rank = 18) of the tomato canopy at different time points. During the morning period, the upper canopy in the N–S orientation showed higher values of solar radiation interception than the E–W orientation. When the sun reaches its highest point in the sky well around noon, the E–W orientation performed better on light interception and uniformity of both upper- and middle-layer canopy. This effect also corresponds to Figure 8C, during midday (11:00–13:00), the horizontal direction of sunlight is shining directly from south to north, which is directly in line with N–S canopy orientation, and which will cause more plant self-shading than the E–W orientation. As the sun went down, the light performance of both the orientations was roughly the same at 4 o’clock late afternoon.

### Tomato Plant Architecture

**FIGURE 10:**
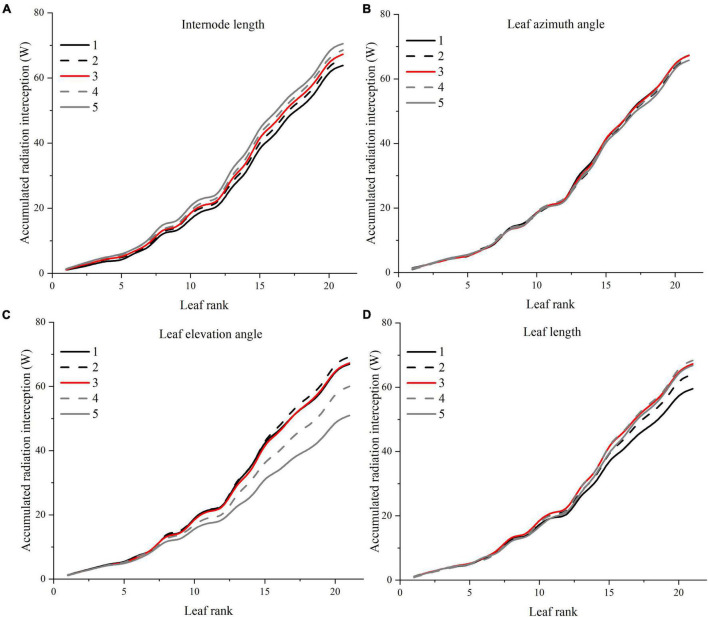
Effects of different leaf architectural shapes on accumulated radiation interception as leaf rank increases. Simulated for the canopy located at the middle of the greenhouse to avoid any border effects. E.g., internode length **(A)**, leaf azimuth angle **(B)**, leaf elevation angle **(C)**, and leaf length **(D)**.

**FIGURE 11:**
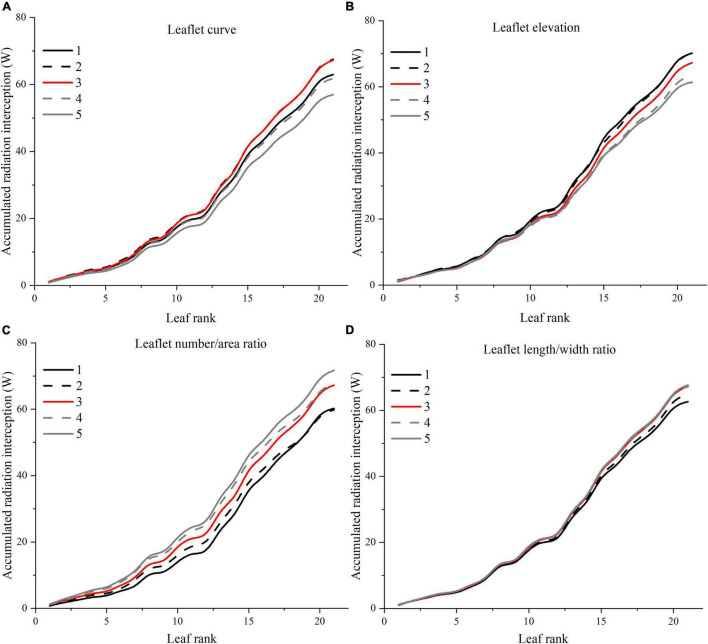
Effects of different leaflet architectural shapes on accumulated radiation interception as leaf rank increases. Simulated for the canopy located at the middle of the greenhouse to avoid any border effects. E.g., leaflet curve **(A)**, leaflet elevation **(B)**, leaflet number/area ratio **(C)**, and leaflet length/width ratio **(D)**.

**FIGURE 12:**
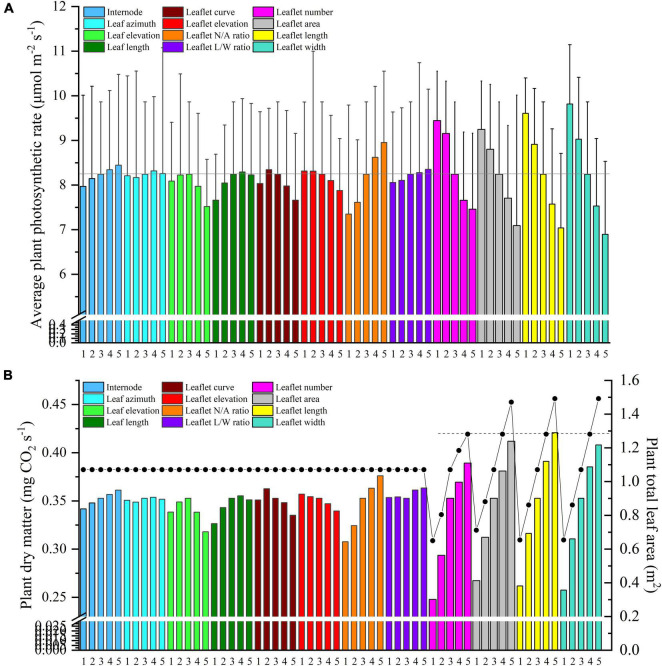
Midday plant average photosynthesis **(A)** and dry matter **(B)** of different leaf and leaflet level architectural shapes under the homogeneous canopy (planting pattern 1) of N–S orientation with *P* = 0.65 m and *S* = 0.3 m.

A visual 3D reconstruction of the four leaf-level architecture shapes and four leaflet-level architecture shapes of tomato crop used for simulation is shown in Figures 3, 4, respectively. The above-mentioned different morphologies of tomato crops were then simulated individually by the homogeneous canopy (planting pattern 1) under N–S orientation with *p* = 0.65 m and *S* = 0.3 m (Figure 2). To further reduce the influence of greenhouse east and west side wall structure, the accumulated solar radiation interception variabilities of each leaf rank of the whole middle part canopy (−5 to 5 m to the middle of greenhouse length) were averaged and shown in Figures 10, 11. The canopy photosynthesis and dry matter performance of each architecture shape adaptation was calculated and depicted in Figure 12.

#### Internode Length

Light interception and photosynthesis are generally positively affected by the internode length, and an increase in the internode length by 4 cm (from R−2 to R + 2 cm) lead to an increased plant cumulative light interception by nearly 10.5% inside the canopy (Figure 10A), also resulting in an increase of canopy photosynthesis by 5.9% (Figure 12A).

#### Leaf Azimuth Angle

Leaf azimuth angle, also known as leaf phyllotactic angle, is defined as the angle between two adjacent leaves along the main plant stem, with a common value of the “Golden angle” 137.5° (Thompson, 1992; Atherton and Rudich, 2012). The reference structure, which is near the “Golden angle,” appears to have the best radiation performance, and changes in the leaf azimuth angle hardly affected light interception (Figure 10B). It also had limited influence on photosynthesis and dry matter (Figure 12). But an increase in the leaf azimuth angle to 180° will eventually decrease the plant cumulative light interception by nearly 2.2% inside the canopy (Figure 10B).

#### Leaf Elevation Angle

The highest light interception was achieved with the leaf elevation angle of 30°. The canopy light interception of reference angle 0° was similar to the 60° angle and ranked second. However, the 30° leaf elevation angle was similar to the reference structure (0°) on the plant average photosynthesis performance (with higher standard error), and surprisingly lower on the dry matter. This phenomenon may be explained by the fact that although the upper part of the canopy (rank 15–21) of 30° elevation angle adaptation intercepted higher amount of radiation (Figure 10C), the northern part of the canopy still gets lower amount of radiation compared to the reference structure due to the shading effects of higher elevation angle. With the reduction of reference elevation angle, the cumulative light interception decreased dramatically by 32%, resulting in photosynthesis dropping by 8.7%. Overall, results showed that the reference leaf elevation angle of 0° is already the ideal angle.

#### Leaf Length

As Figure 10D shows, leaf length at 1.2 × *R* value has the highest light interception. Shortening the leaf length from reference value will only reduce the light interception by 4.9 and 11.5%. Similar patterns have also been shown on photosynthesis and dry matter performance (7.1% reduction) of leaf length (Figure 12).

#### Leaflet Curve

An increase in leaflet curvature from completely flat leaflets (0 × Z_*R*_) to the most curved adaptation (3 × Z_*R*_) resulted in a decrease in light absorption by 15.6% (Figure 11A). Increasing the leaflet curvature in the opposite direction (−2 × Z_*R*_) will also cause the canopy light interception to drop by about 6.7%. Similar result patterns have also been found on the canopy photosynthesis and dry matter performance (Figure 12). From the simulations, we can now infer that more flatten leaflets tend to intercept more radiation.

#### Leaflet Elevation

Leaflet elevation at the highest angle (60°) intercepted the most radiation, then comes to the 30° angle value ranked the second (0.7% reduction on dry matter). Leaflet only intercept more radiation when the leaflet elevated at certain angles, and the changes in the total amount of intercepted solar radiation were not significant (Figure 11B). The average canopy photosynthesis and dry matter follow a linearly decreasing pattern when the leaflet elevation angle decreases (Figure 12).

#### Leaflet Number/Area Ratio

Based on the results of the simulation, the canopy showed that photosynthesis was strongly affected by the leaflet N/A ratio (Figure 12A). With the plant total leaf area staying the same, by increasing the leaflet N/A ratio, the middle and upper part of the plant will be less shaded than the reference structure (causing an increase in radiation by 6.7%). Conversely, the reduction of leaflet N/A ratio will cause a drop in the light interception of the middle part of the plant (causing a reduction in radiation by 10.5%) (Figure 11C). Similar patterns also showed on the average photosynthetic rate with a increment of 7.6% and has the most potent effects on the canopy dry matter (6.6%) among other architecture scenarios with same plant leaf area.

#### Leaflet Length/Width Ratio

The plant with thinner longer leaves (1.96 × R) will intercept slightly more radiation than the reference structure (Figure 11D) with a 0.6% increment on radiation,1.3% on photosynthesis, and 3% on dry matter compared to the reference value. On the other hand, plants with broader leaves (0.36 × R) exhibit the opposite phenomenon, decreasing dramatically on the total light interception (6.9%). The average photosynthetic rate dropped as the leaves got wider than the reference value (2.2%), yet the dry matter was roughly the same with reference structure (Figure 12).

#### Changing Plant Leaf Area

Changing the plant total leaf area can be done in four ways, including increasing the leaflet number, growing leaflet area proportionally, and expanding the leaflet length or leaflet width (Supplementary Figure 2). As the results of the simulation showed, the canopy photosynthesis was strongly affected by the leaflet width (Figure 11A). Decreasing the leaflet width will further cause photosynthesis to increase dramatically, and this phenomenon further proves that increasing the leaf L/W ratio will have a positive influence on the plant photosynthesis. Figure 12B shows these four ways with the same leaflet area increment, which marked with a dashed line, led to a rise in the dry matter by 10.3% (leaflet number scenario), 8% (leaflet area scenario), 10.9% (leaflet length scenario), and 9.2% (leaflet width scenario) correspondingly. Increasing the leaflet length thus proved to be the most effective way of increasing the canopy photosynthesis and dry matter. This result is in agreement with Sarlikioti et al. (2011a).

#### Ideal Tomato Plant Architecture

**FIGURE 13:**
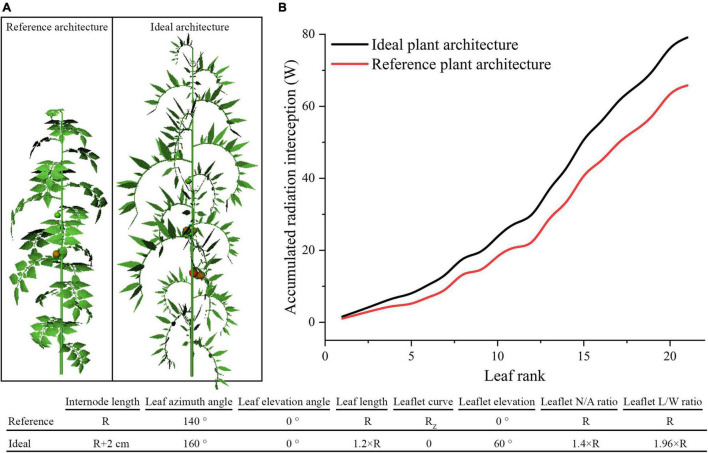
The ideal tomato plant architecture which combined all leaf- and leaflet level optimal traits **(A)**. Comparison of ideal and reference plant architecture on canopy accumulated radiation interception as leaf rank increases **(B)**.

According to previous assessment of tomato plant architecture, the ideal tomato plant architecture was further designed to accomplish the best light absorption performance, which was suitable for homogeneous canopy (planting pattern 1) under N–S orientation. As shown in Figure 13, the optimal parameter value of each leaf- and leaflet-level architecture shape adaptation were selected and combined to form the ideal tomato plant architecture. In Figure 13B, the ideal architecture shape exhibited a significant increase in light interception of 20.2% compared to the reference structure.

### Partial Least Squares Path Modeling Analysis

The PLS–PM analysis was performed to understand how the above simulated large-scale dataset of different scenarios were related to each other and to the inner causative relationship (Granier and Vile, 2014). In the path modeling process, each latent value (e.g., planting strategy) is a composite value of its associate simulated variables (determined through correlations) and so formed the outer model (with the outer loadings please refer to Supplementary Table 1). The inner model allows us to study the connections of latent values, which are represented by path coefficients (Supplementary Table 2), with *R*^2^ values indicating the degree to how well the latent value is represented by other endogenous latent values (Supplementary Table 3).

**FIGURE 14:**
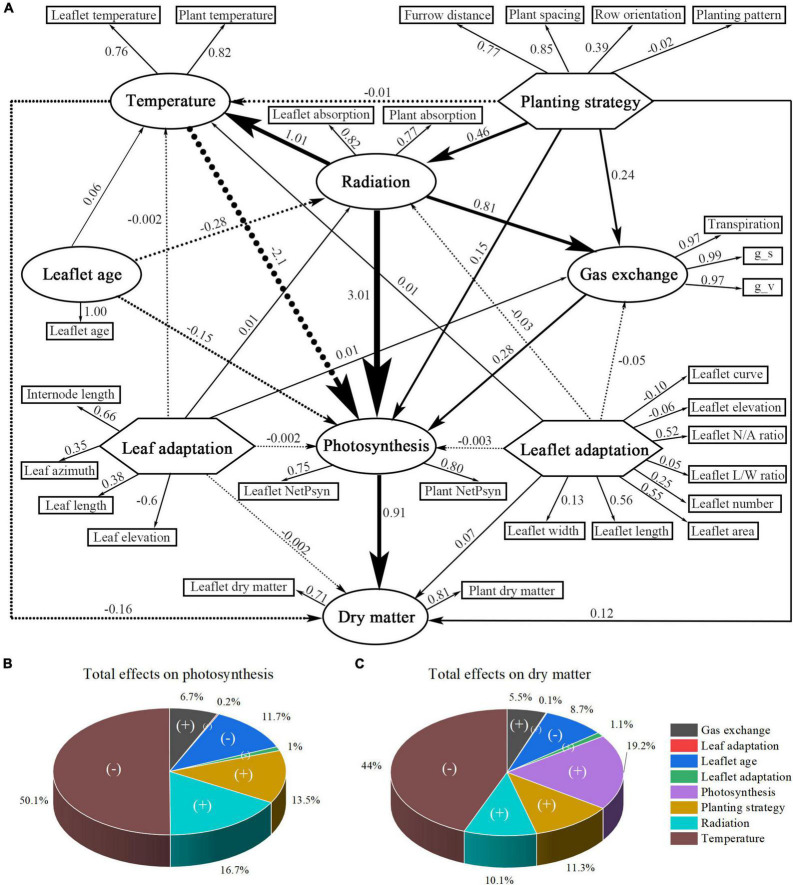
Partial least-squares path modeling (PLS-PM) of all collected physiological and morphological data **(A)**. The finalized version of the PLS-PM is shown. Simulated variables are represented in a rectangle form, while traits within the big circles and polygons are latent variables. Indicated are the loadings (the correlations between a latent variable and its simulated variables) and the path coefficients calculated after 1,000 bootstraps. Total effects of different latent values on the outputs of the PLS-PM: photosynthesis **(B)** and yield **(C)**. (+) indicates a positive effect while (–) indicates a negative effect. Percentages are the proportion of path weights contributing to each output.

The model indicates that the planting strategy has a strong positive influence on radiation, gas exchange, and photosynthesis, with an indirect effect (1.415) of planting strategy on photosynthesis that was mediated by the radiation (Supplementary Table 4). The planting strategy is well-represented by the simulated variable plant spacing (0.85), furrow distance (0.77), and row orientation (0.39). The planting pattern, on the other hand, has a very limited impact on representing the planting strategy (−0.02) (Figure 13). The modeling result also showed that increasing the values of leaf-level adaptation will have a positive impact on radiation (0.01) and a negative impact on temperature (−0.002), photosynthesis (−0.002), and dry matter (−0.002). The impact of leaf-level plant architecture-related adaptations was minimal, and therefore negligible. On the other hand, leaflet-level adaptations have a relatively larger effect, and increasing the values of leaflet-level adaptation will have a negative influence on the radiation (−0.03), photosynthesis (−0.003), and yet positive effects on temperature (0.01) and dry matter (0.07). This result corresponds well with our former analysis result. However, leaf- and leaflet-level architectural shapes both have minor impacts on the overall plant radiation, temperature, photosynthesis, and dry matter (Figure 14A); compared to leaf-level adaptation (0.027), leaflet-level adaptation has a relatively more substantial indirect effect (−0.101) on photosynthesis that was mediated by the radiation (Supplementary Table 4).

Figures 14B,C displays the effect of each trait on the overall output of the plants (photosynthesis and dry matter). Radiation and temperature show the most significant influence on photosynthesis and dry matter, followed by the plant strategy being the third and the positive contributor to photosynthesis and dry matter. Leaf-level and leaflet-level architectural shapes both have relatively small influence compared with planting strategy.

## Discussion

The primary focus of greenhouse planting and breeding have been on either the planting strategy or the plant architecture, respectively. The non-uniformity of the microclimate inside solar greenhouse has been relatively ignored in the actual practice and breeding efforts. However, these traits are interconnected and therefore must be considered at the same time. In the current study, we investigated the role and level of significance of four kinds of planting strategies and eight different organ-level plant architectures by simulating the actual planting process in the greenhouse and considering the greenhouse microclimate.

The data showed that between the four kinds of planting strategies (plant spacing, furrow distance, row orientation, planting pattern), increasing the plant spacing will be the most effective way to increase the light interception and photosynthesis rate. This outcome has also been proven by the PLS–PM analysis. The furrow distance increment, on the other hand, is more effective under E–W orientation. E–W orientation performed better on light interception but weaker on light uniformity, with similar outcome been given by van der Meer et al. (2021) on hedgerow tomato crops. N–S orientation has a better light interception in the morning and afternoon and is weaker at midday (Figure 9). Although the planting patterns have limited influence on the total amount of light interception (−0.02, through planting strategy, Figure 14), slightly better light uniformity was still found in planting pattern 4 (incrementing row). Therefore, it is better to use E–W orientation of planting pattern 4 which, is more suitable for mechanized planting, during the actual planting process.

From the plant architecture results of this work, we can conclude that leaflet number/area ratio may be among the best-investigated traits. It has shown that incrementing on the N/A ratio will significantly increase the amount of light captured by plants compared to the other investigated traits of leaf- or leaflet-level adaptations. This study also showed that tomato with longer thinner leaflet has better photosynthesis and dry matter performance. Among all leaf-and leaflet-level architectural shapes with the same plant leaf area, increasing the leaflet number, and at the same time shrink the leaflet area is the best solution. In other words, many-small leaves perform better than fewer-larger leaves. The best architectural form for the leaf and leaflet elevation angle is 0 and 60°, and other scenarios dropping on the leaf and leaflet elevation angle will mainly cause a decrease in the photosynthesis and dry matter; several studies have shown that changes in the elevation angles will significantly influence light capture in different environments (Valladares and Pearcy, 1998; Niinemets and Fleck, 2002). Modification on the leaf azimuth angle results in no improvement in photosynthesis and dry matter. This finding agrees with Sarlikioti et al. (2011a). The ideal tomato plant architecture which combined all above optimal traits was simulated and results in a significant increase on the light interception performance of the canopy. This combination of optimal architectural traits could potentially increase the yield. The presented ideal structure information could, in turn, be used as a guidance for modern plant breeding procedures, thereby eluding the breeding efforts toward tomato phenotypic traits with optimal light interception.

The result of PLS–PM showed that leaf temperature has the strongest most substantial negative effect on photosynthesis. This phenomenon is caused by the fact that at midday, the greenhouse CO_2_ concentration (C_*a*_) is 331 ppm, under which the most suitable temperature for best net photosynthesis performance is around 25°C; thus any rise or deduction in the leaflet temperature will reduce the maximum photosynthesis capability; therefore, according to the analysis, it is recommended to use CO_2_ fertilization inside the greenhouse during the day to further decrease the biochemical capacity limitation caused by the temperature (Zhang Y. et al., 2021). The second trait is radiation, which is strongly affected by the planting strategy (0.46) and has a strong positive effect on temperature (1.01), gas exchange (0.81), and photosynthesis (3.01). This was followed by the planting strategy, which was ranked third for photosynthesis and fourth for dry matter, and in which the plant spacing is the most effective way of increasing the light interception. The leaflet age is also a major negative effect that limits the photosynthesis and dry matter, in which the 20-day old leaflet is the most suitable age for the maximum leaf photosynthesis, and any subsequent older leaflets will reduce the photosynthesis capability. However, the plant architecture has a relatively small impact on dry matter compared to planting strategy, which is still enough to make a difference when it comes to the accumulation of the entire growing season.

Further investigations are needed to investigate different developmental stages or the transferability of the results to other locations (latitudes), which both were not part of the presented investigation and would have transcended the limits of possible simulations.

## Conclusion

The importance of greenhouse planting strategy and plant architecture for tomato light interception, temperature, photosynthesis, and dry matter was investigated in detail using the virtual solar greenhouse and tomato plant. The PLS–PM approach was employed to analyze the causative relationships. Our study led to the conclusion that the planting strategies have a more significant impact overall on plant radiation, temperature, photosynthesis, and dry matter compared to plant architecture changes, among which plant spacing has the most significant impact. The planting pattern surprisingly does not have a considerable effect on the canopy light interception but rather light uniformity. We also showed that increasing the plant leaflet N/A ratio will have a considerable effect on the dry matter compared to other architectural traits. Increasing the internode length, leaf length, leaflet L/W ratio, and the leaflet elevation angle will also have a positive influence on the plant dry matter. The ideal tomato plant architecture was also designed to provide information on phenotypic traits selection of breeding process. Our model and analysis methods may also be directly transferred to other plant species and different types of greenhouses with only minor modifications. Therefore, the presented method has proven to provide new insights into greenhouse canopy management and the breeding process to optimize the planting strategy and plant architecture. The proposed model can be directly used to simulate other plant species or greenhouse shapes with only a few adaptations that makes it a valuable tool for future investigations.

## Data Availability Statement

The raw data supporting the conclusions of this article will be made available by the authors, without undue reservation.

## Author Contributions

YZ, MH, XL, and TL planned and designed the research. YZ performed experiments and conducted fieldwork. MH provided methodology and software. YZ and MH implemented the models, analyzed the data, and wrote the manuscript. MH, YL, DX, AL, XL, and TL reviewed and edited the manuscript. XL acquired the funding. TL supervised the project. All authors contributed to the article and approved the submitted version.

## Conflict of Interest

The authors declare that the research was conducted in the absence of any commercial or financial relationships that could be construed as a potential conflict of interest.

## Publisher’s Note

All claims expressed in this article are solely those of the authors and do not necessarily represent those of their affiliated organizations, or those of the publisher, the editors and the reviewers. Any product that may be evaluated in this article, or claim that may be made by its manufacturer, is not guaranteed or endorsed by the publisher.
