# Supplementary material for: Analyzing the Impact of Greenhouse Planting Strategy and Plant Architecture on Tomato Plant Physiology and Estimated Dry Matter
Source: Front Plant Sci. 2022 Feb 15;13:828252. doi: 10.3389/fpls.2022.828252 (PMC8885523; doi:10.3389/fpls.2022.828252)
Supplement: Supplementary file 1 [file Data_Sheet_1.docx]

Supplementary Material

# Supplementary Figures


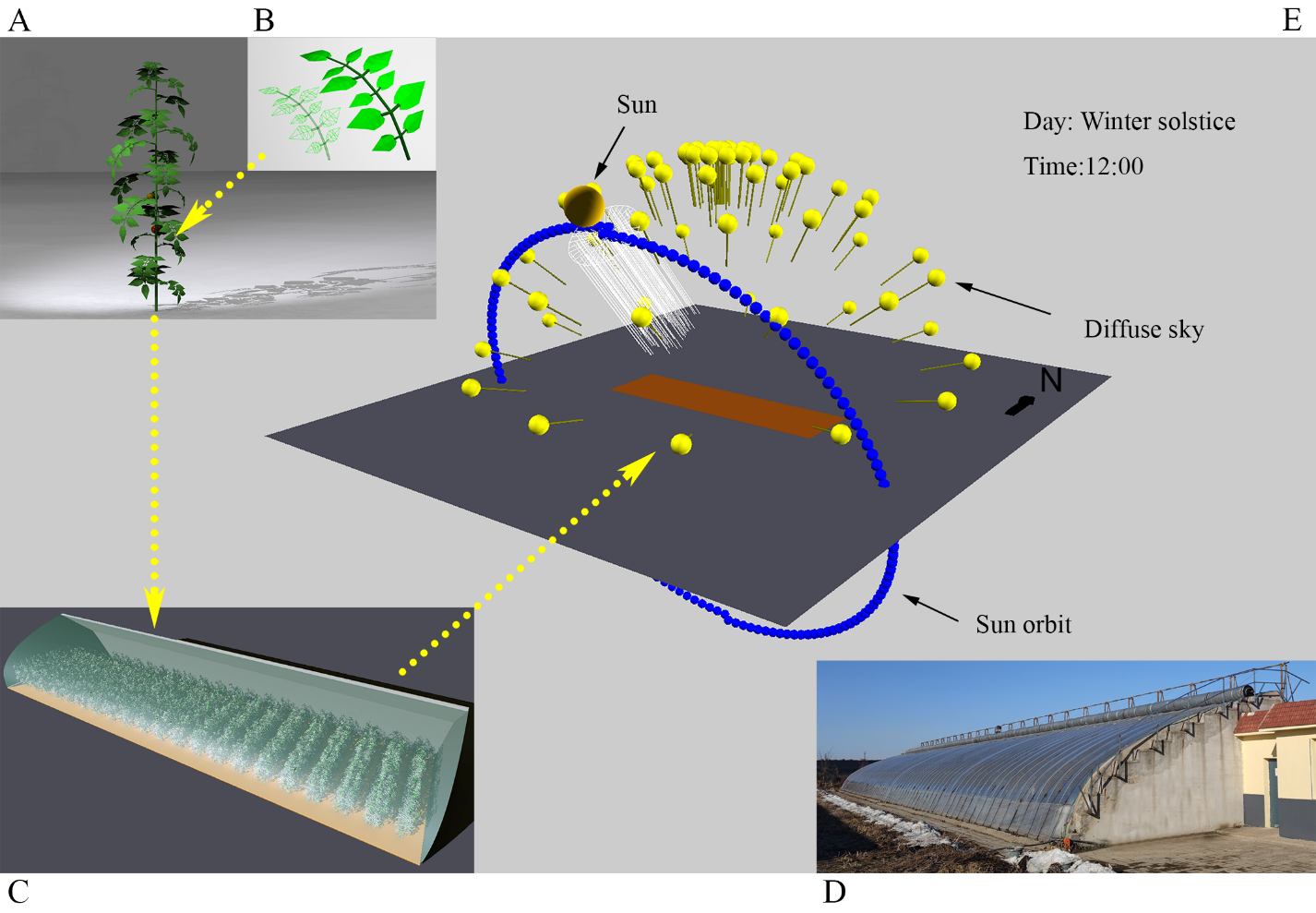


**Supplementary Figure S1.** Rendered image of a single, adult tomato plant as used in the canopy (a). A detailed enlargement of a single leaf as well as wireframe visualization of the underlying triangulated mesh structure of the leaflets (b). (c) Snapshot of the 3D model of a 30 m long Liaoshen-solar greenhouse (LSG) including the tomato canopy. (d) Photograph of a real LSG at Shenyang Agricultural University (photograph by Yue Zhang). (e) Visualization of the sun, sky, and greenhouse model. The yellow spheres represent the 72 direct light sources of the diffuse sky model. The big orange sphere and the white lines indicate the sun and the direct light rays emitted by the sun. The blues spheres are visual track marks of sun movement over one day (day 355 of the year).


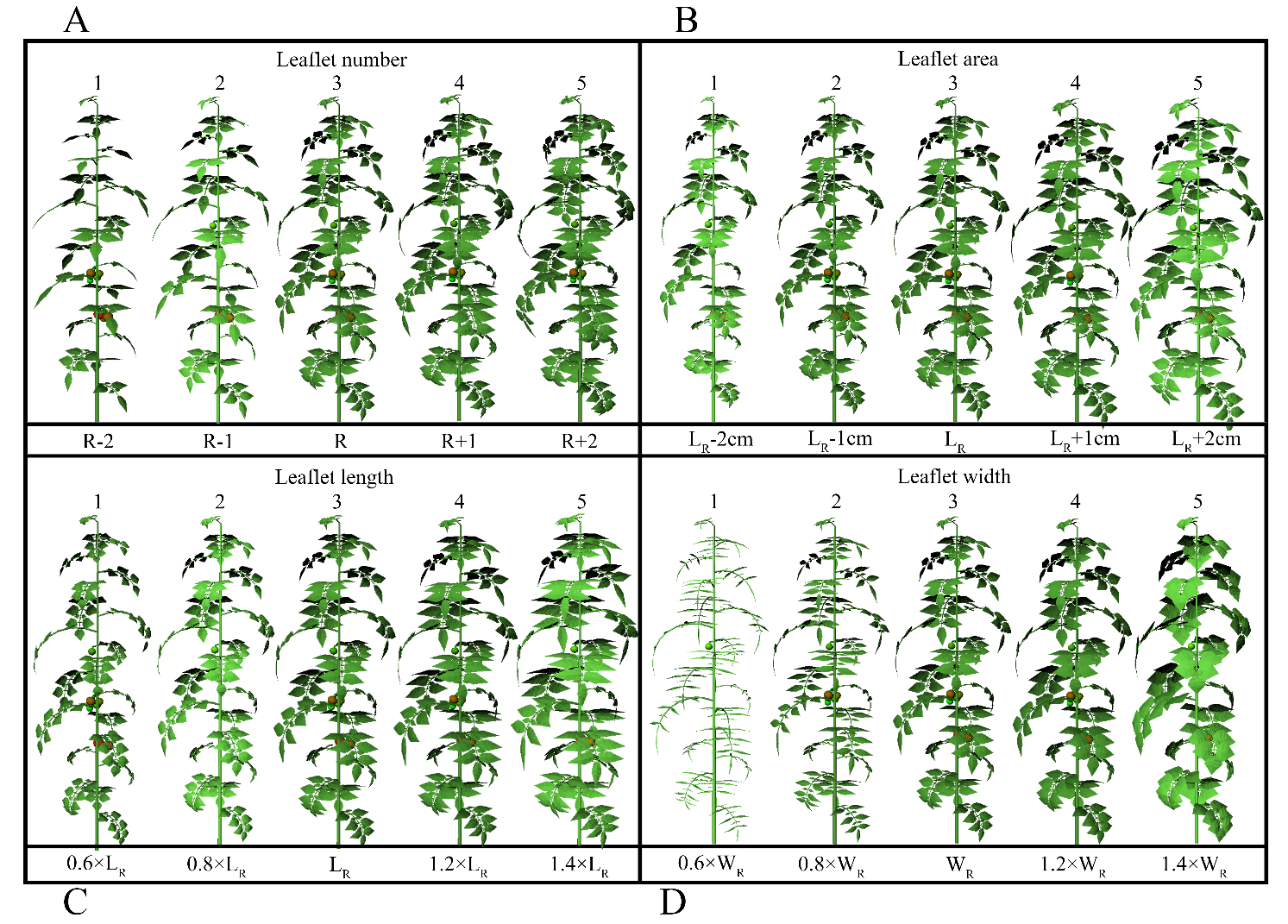


**Supplementary Figure S2.** Detailed configurations of the changing plant leaf area architectural traits. Each plant architecture group was simulated individually with 5 adaptations for each scenario. Each adaptation was applied to every leaf rank of tomato plant, with visual 3D representation of each leaf level adaptation, namely: leaflet number (R-2, R-1, R, R+1, R+2) **(A)**, leaflet area in proportion (L_R_-2 cm, L_R_ -1 cm, L_R_ cm, L_R_+1 cm L_R_+2 cm) **(B)**, leaflet length (0.6×L_R_, 0.8×L_R_, L_R_, 1.2×L_R_, 1.4×L_R_) **(C)**, and leaflet width (0.6×W_R_, 0.8×W_R_, W_R_, 1.2×W_R_, 1.4×W_R_) **(D)**. L_R_ represent the reference value of leaflet length. W_R_ being the reference value of leaflet width.


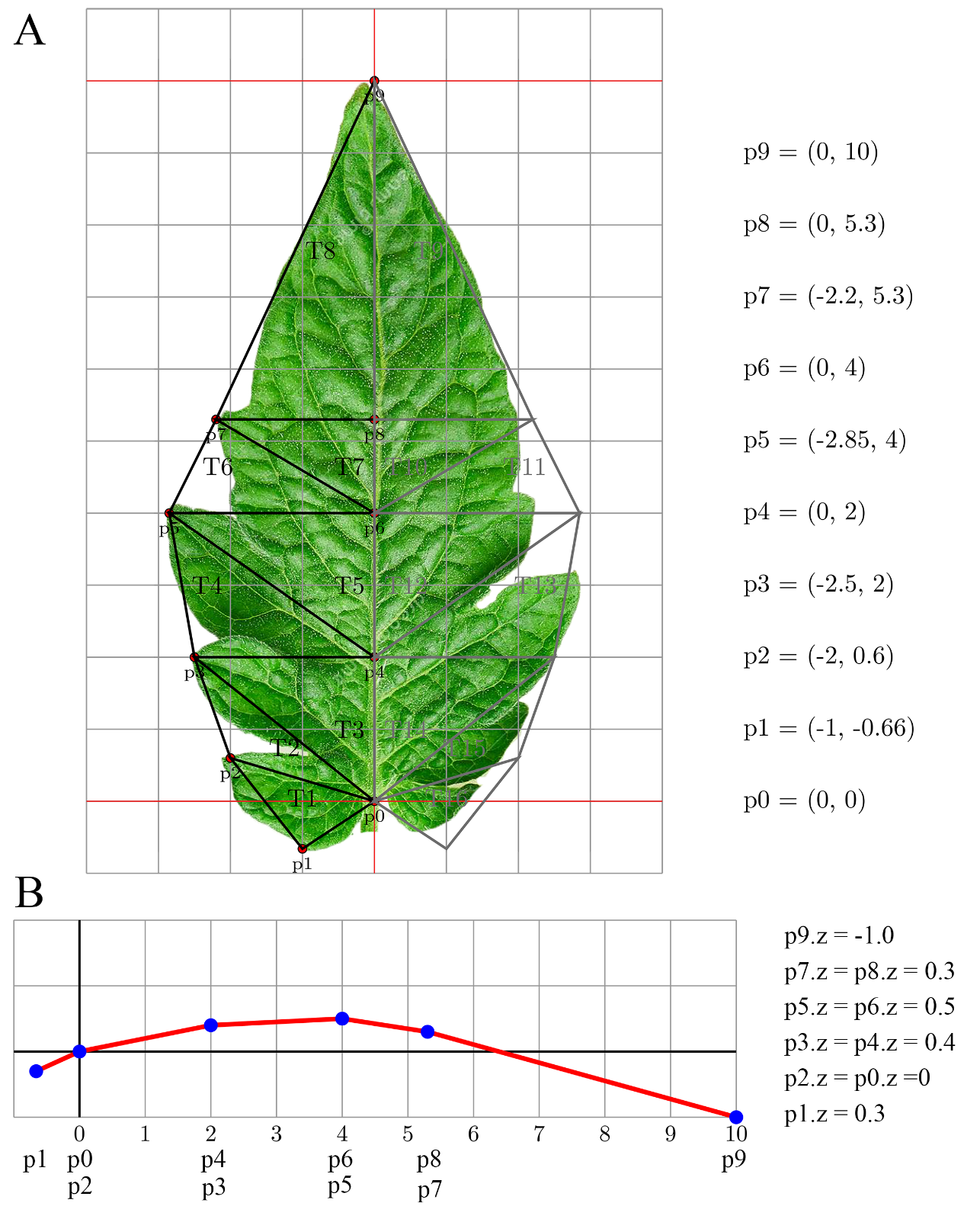


**Supplementary Figure S3.** Detailed modeling process of tomato leaflet shape. p0 to p9 represent the modelling point of left half leaflet. Leaflet curvature was defined using the Z axis value of each point Z_R_ represent the measured reference value of each point. The different leaflet curvature adaptations were achieved by changing the Z axis value of each point and then rescaled according to the reference length of the leaflet.

**
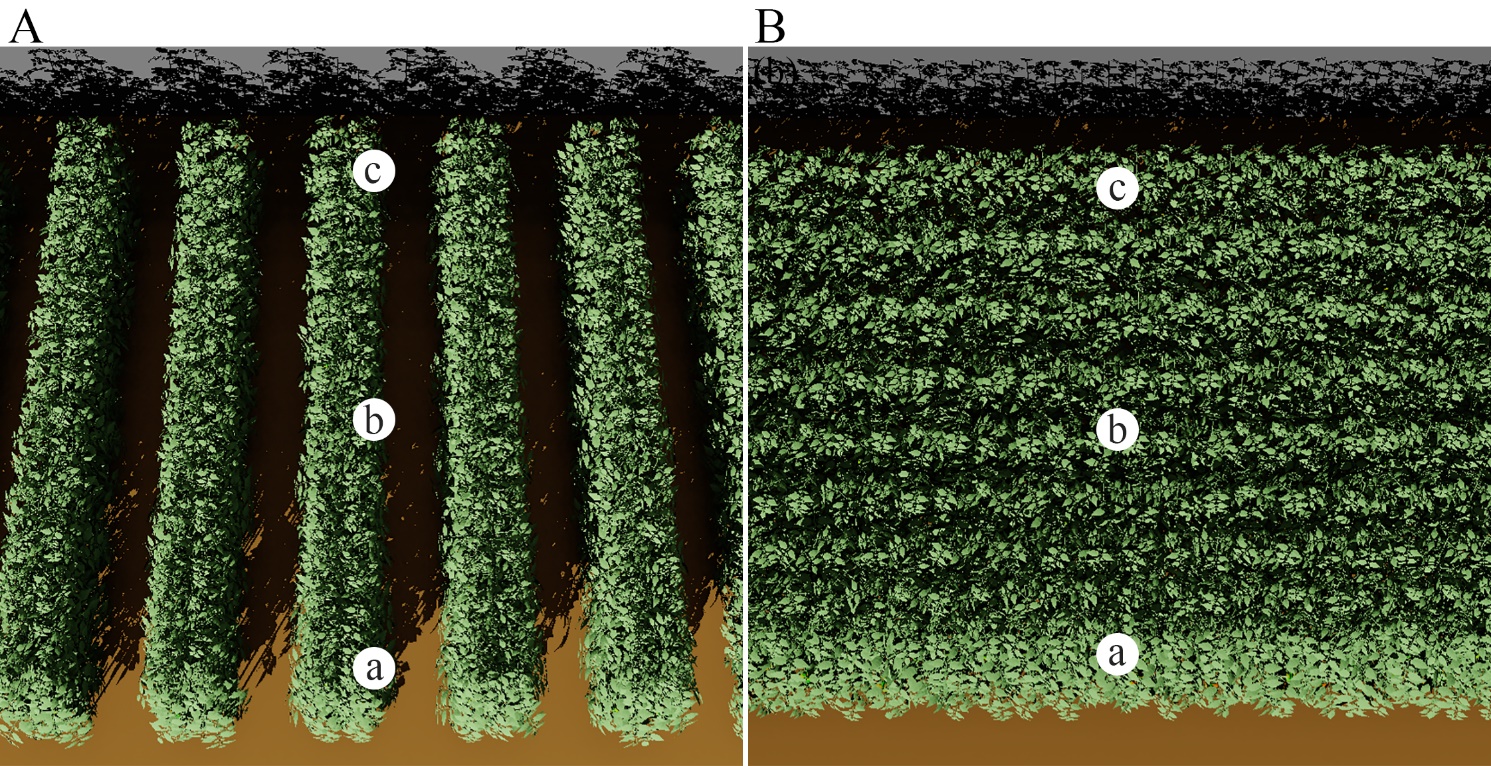
**

**Supplementary Figure S4.** Snapshot of the 3D model of the planting pattern 4 (incrementing row) of N-S (a) and E-W (b) orientation.

**
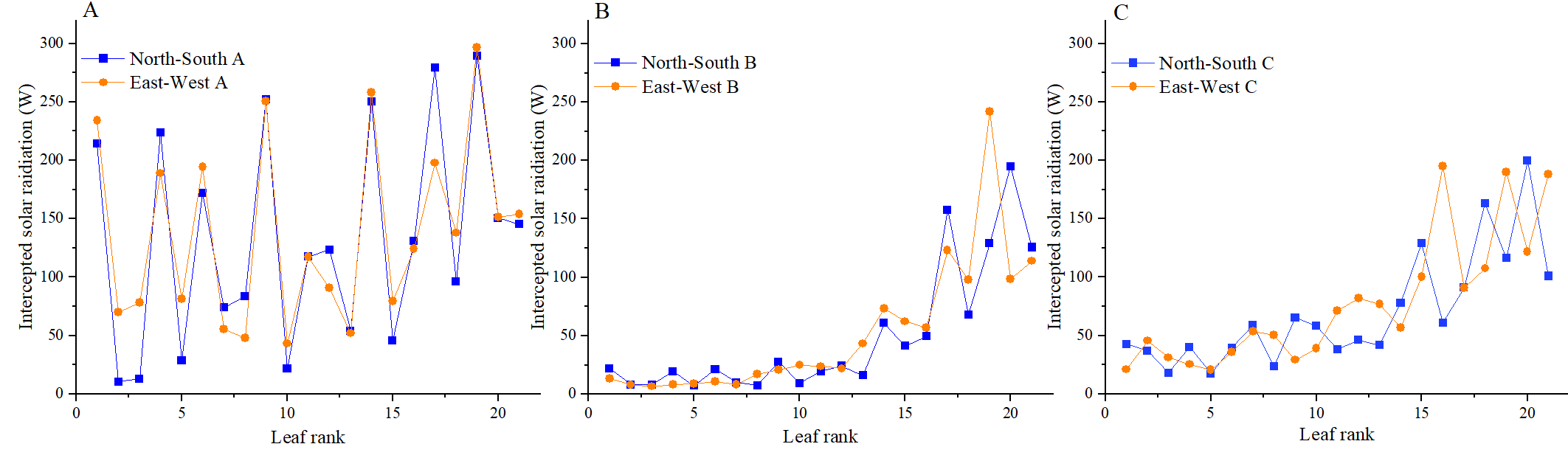
**

**Supplementary Figure S5.**  Comparison of plant intercepted solar radiation located at point a (A), b (B), and c (C) point of N-S orientation and E-W Orientation of planting pattern 4 (incrementing row).

# Supplementary Tables

**Supplementary Table.S1** Outer model loadings and significance. Original and sample mean for each simulated variable are shown, with standard deviation and P-values.

|  | Original Sample (O) | Sample Mean (M) | Standard Deviation (STDEV) | P Values |
| --- | --- | --- | --- | --- |
| Age <- Leaflet age | 1 | 1 | 0 | 0 |
| Leaflet absorption <- Radiation | 0.821 | 0.821 | 0 | 0 |
| Plant_dry matter <- Dry matter | 0.806 | 0.806 | 0.001 | 0 |
| Leaflet_dry matter <- Dry matter | 0.71 | 0.71 | 0.001 | 0 |
| Furrow distance <- Planting strategy | 0.77 | 0.77 | 0.001 | 0 |
| Internode <- Leaf adaptation | 0.661 | 0.656 | 0.049 | 0 |
| Leaf azimuth <- Leaf adaptation | 0.035 | 0.035 | 0.064 | 0 |
| Leaf elevation <- Leaf adaptation | -0.632 | -0.63 | 0.051 | 0 |
| Leaf length <- Leaf adaptation | 0.403 | 0.399 | 0.057 | 0 |
| Leafet elevation <- Leaflet adaptation | -0.061 | -0.061 | 0.008 | 0 |
| Leaflet area <- Leaflet adaptation | 0.559 | 0.559 | 0.007 | 0 |
| Leaflet L/W ratio <- Leaflet adaptation | 0.055 | 0.055 | 0.007 | 0 |
| Leaflet N/A ratio <- Leaflet adaptation | 0.522 | 0.521 | 0.027 | 0 |
| Leaflet number <- Leaflet adaptation | 0.253 | 0.252 | 0.038 | 0 |
| Leaflet width <- Leaflet adaptation | 0.135 | 0.134 | 0.007 | 0 |
| Leaflet curve <- Leaflet adaptation | -0.102 | -0.102 | 0.007 | 0 |
| Leaflet length <- Leaflet adaptation | 0.559 | 0.558 | 0.006 | 0 |
| Leaflet temperature <- Temperature | 0.751 | 0.751 | 0 | 0 |
| Leaflet NetPsyn <- Photosynthesis | 0.753 | 0.753 | 0 | 0 |
| Plant absorption <- Radiation | 0.767 | 0.767 | 0 | 0 |
| Plant NetPsyn <- Photosynthesis | 0.794 | 0.794 | 0 | 0 |
| Plant temperature <- Temperature | 0.822 | 0.822 | 0 | 0 |
| Plant spacing <- Planting strategy | 0.853 | 0.853 | 0 | 0 |
| Planting pattern <- Planting strategy | -0.019 | -0.019 | 0.001 | 0 |
| Row orientation <- Planting strategy | 0.39 | 0.39 | 0.001 | 0 |
| Transpiration <- Gas exchange | 0.972 | 0.972 | 0 | 0 |
| g_s <- Gas exchange | 0.985 | 0.985 | 0 | 0 |
| g_v <- Gas exchange | 0.973 | 0.973 | 0 | 0 |

**Supplementary Table.S2** The path coefficients between the latent variables. Original and sample mean between each two latent variables are shown, with standard deviation and P-values.

|  | Original Sample (O) | Sample Mean (M) | Standard Deviation (STDEV) | P Values |
| --- | --- | --- | --- | --- |
| Gas exchange -> Photosynthesis | 0.284 | 0.283 | 0.001 | 0 |
| Leaf adaptation -> Dry matter | -0.002 | -0.002 | 0 | 0 |
| Leaf adaptation -> Gas exchange | 0.01 | 0.01 | 0.001 | 0 |
| Leaf adaptation -> Photosynthesis | -0.002 | -0.002 | 0 | 0 |
| Leaf adaptation -> Radiation | 0.009 | 0.009 | 0.001 | 0 |
| Leaf adaptation -> Temperature | -0.002 | -0.002 | 0 | 0 |
| Leaflet adaptation -> Dry matter | 0.067 | 0.067 | 0.002 | 0 |
| Leaflet adaptation -> Gas exchange | -0.048 | -0.048 | 0.001 | 0 |
| Leaflet adaptation -> Photosynthesis | -0.003 | -0.003 | 0 | 0 |
| Leaflet adaptation -> Radiation | -0.033 | -0.033 | 0.001 | 0 |
| Leaflet adaptation -> Temperature | 0.014 | 0.014 | 0 | 0 |
| Leaflet age -> Photosynthesis | -0.156 | -0.156 | 0 | 0 |
| Leaflet age -> Radiation | -0.282 | -0.282 | 0.001 | 0 |
| Leaflet age -> Temperature | 0.064 | 0.064 | 0 | 0 |
| Photosynthesis -> Dry matter | 0.907 | 0.906 | 0.001 | 0 |
| Planting strategy -> Dry matter | 0.125 | 0.125 | 0.001 | 0 |
| Planting strategy -> Gas exchange | 0.242 | 0.242 | 0.001 | 0 |
| Planting strategy -> Photosynthesis | 0.154 | 0.154 | 0 | 0 |
| Planting strategy -> Radiation | 0.463 | 0.463 | 0.001 | 0 |
| Planting strategy -> Temperature | -0.009 | -0.009 | 0 | 0 |
| Radiation -> Gas exchange | 0.808 | 0.808 | 0 | 0 |
| Radiation -> Photosynthesis | 3.054 | 3.053 | 0.005 | 0 |
| Radiation -> Temperature | 1.005 | 1.005 | 0 | 0 |
| Temperature -> Dry matter | -0.163 | -0.163 | 0.001 | 0 |
| Temperature -> Photosynthesis | -2.109 | -2.109 | 0.005 | 0 |

**Supplementary Table.S3** R^2^ values for each Latent Variable in the inner model. Original and sample mean values R^2^ values.

| LV | Original Sample (O) | Sample Mean (M) | Standard Deviation (STDEV) | P Values |
| --- | --- | --- | --- | --- |
| Dry matter | 0.693 | 0.693 | 0 | 0 |
| Gas exchange | 0.535 | 0.535 | 0 | 0 |
| Photosynthesis | 0.928 | 0.928 | 0 | 0 |
| Radiation | 0.295 | 0.295 | 0.001 | 0 |
| Temperature | 0.969 | 0.969 | 0 | 0 |

**Supplementary Table.S4** Specific indirect effects between the latent variables. Original and sample mean for each indirect path are shown, with standard deviation and P-values.

|  | Original Sample (O) | Sample Mean (M) | Standard Deviation (STDEV) | P Values |
| --- | --- | --- | --- | --- |
| Radiation -> Gas exchange -> Photosynthesis | -0.229 | -0.229 | 0.001 | 0 |
| Leaflet age -> Temperature -> Photosynthesis | -0.138 | -0.138 | 0 | 0 |
| Leaf adaptation -> Radiation -> Gas exchange -> Photosynthesis -> Dry matter | -0.002 | -0.002 | 0 | 0 |
| Planting strategy -> Radiation -> Gas exchange -> Photosynthesis | 0.106 | 0.106 | 0 | 0 |
| Radiation -> Gas exchange -> Photosynthesis -> Dry matter | 0.208 | 0.208 | 0.001 | 0 |
| Leaf adaptation -> Radiation -> Photosynthesis -> Dry matter | 0.024 | 0.024 | 0.002 | 0 |
| Radiation -> Temperature -> Photosynthesis | -2.121 | -2.12 | 0.005 | 0 |
| Planting strategy -> Temperature -> Dry matter | 0.002 | 0.002 | 0 | 0 |
| Planting strategy -> Radiation -> Temperature | 0.466 | 0.466 | 0.001 | 0 |
| Leaflet adaptation -> Radiation -> Gas exchange | -0.027 | -0.027 | 0.001 | 0 |
| Leaflet adaptation -> Temperature -> Photosynthesis -> Dry matter | -0.028 | -0.027 | 0.001 | 0 |
| Leaf adaptation -> Radiation -> Photosynthesis | 0.027 | 0.027 | 0.002 | 0 |
| Leaflet adaptation -> Radiation -> Gas exchange -> Photosynthesis -> Dry matter | 0.007 | 0.007 | 0 | 0 |
| Leaflet adaptation -> Gas exchange -> Photosynthesis | 0.014 | 0.014 | 0 | 0 |
| Leaflet adaptation -> Gas exchange -> Photosynthesis -> Dry matter | 0.012 | 0.012 | 0 | 0 |
| Leaf adaptation -> Temperature -> Photosynthesis -> Dry matter | 0.004 | 0.004 | 0 | 0 |
| Leaf adaptation -> Gas exchange -> Photosynthesis -> Dry matter | 0.001 | 0.001 | 0 | 0 |
| Leaflet adaptation -> Radiation -> Gas exchange -> Photosynthesis | 0.008 | 0.008 | 0 | 0 |
| Gas exchange -> Photosynthesis -> Dry matter | 0.257 | 0.257 | 0.001 | 0 |
| Planting strategy -> Radiation -> Temperature -> Dry matter | -0.076 | -0.076 | 0 | 0 |
| Planting strategy -> Temperature -> Photosynthesis | 0.02 | 0.02 | 0 | 0 |
| Leaf adaptation -> Photosynthesis -> Dry matter | -0.002 | -0.002 | 0 | 0 |
| Leaflet adaptation -> Radiation -> Temperature -> Photosynthesis | 0.07 | 0.071 | 0.002 | 0 |
| Leaflet age -> Radiation -> Photosynthesis | -0.861 | -0.861 | 0.002 | 0 |
| Leaflet age -> Temperature -> Photosynthesis -> Dry matter | -0.125 | -0.125 | 0 | 0 |
| Leaf adaptation -> Radiation -> Temperature | 0.009 | 0.009 | 0.001 | 0 |
| Leaflet age -> Radiation -> Temperature | -0.283 | -0.283 | 0.001 | 0 |
| Planting strategy -> Radiation -> Photosynthesis -> Dry matter | 1.283 | 1.282 | 0.003 | 0 |
| Leaf adaptation -> Temperature -> Dry matter | 0 | 0 | 0 | 0 |
| Leaflet age -> Temperature -> Dry matter | -0.011 | -0.011 | 0 | 0 |
| Leaflet age -> Photosynthesis -> Dry matter | -0.141 | -0.141 | 0 | 0 |
| Planting strategy -> Radiation -> Gas exchange -> Photosynthesis -> Dry matter | -0.096 | -0.096 | 0 | 0 |
| Radiation -> Photosynthesis -> Dry matter | 2.769 | 2.768 | 0.005 | 0 |
| Planting strategy -> Temperature -> Photosynthesis -> Dry matter | 0.018 | 0.018 | 0 | 0 |
| Leaflet adaptation -> Temperature -> Photosynthesis | -0.03 | -0.03 | 0.001 | 0 |
| Planting strategy -> Radiation -> Temperature -> Photosynthesis | -0.983 | -0.982 | 0.003 | 0 |
| Leaflet age -> Radiation -> Temperature -> Photosynthesis | 0.598 | 0.598 | 0.002 | 0 |
| Planting strategy -> Radiation -> Temperature -> Photosynthesis -> Dry matter | -0.891 | -0.891 | 0.003 | 0 |
| Leaflet adaptation -> Radiation -> Temperature | -0.033 | -0.033 | 0.001 | 0 |
| Leaf adaptation -> Gas exchange -> Photosynthesis | 0.002 | 0.002 | 0 | 0 |
| Leaflet adaptation -> Radiation -> Photosynthesis | -0.101 | -0.102 | 0.003 | 0 |
| Leaf adaptation -> Radiation -> Temperature -> Dry matter | -0.001 | -0.001 | 0 | 0 |
| Planting strategy -> Radiation -> Photosynthesis | 1.415 | 1.415 | 0.003 | 0 |
| Leaflet age -> Radiation -> Gas exchange -> Photosynthesis -> Dry matter | -0.059 | -0.059 | 0 | 0 |
| Leaf adaptation -> Radiation -> Temperature -> Photosynthesis | -0.019 | -0.019 | 0.001 | 0 |
| Leaflet adaptation -> Temperature -> Dry matter | -0.002 | -0.002 | 0 | 0 |
| Planting strategy -> Gas exchange -> Photosynthesis | 0.069 | 0.069 | 0 | 0 |
| Leaflet adaptation -> Photosynthesis -> Dry matter | -0.003 | -0.003 | 0 | 0 |
| Leaflet age -> Radiation -> Gas exchange -> Photosynthesis | -0.065 | -0.065 | 0 | 0 |
| Leaflet adaptation -> Radiation -> Photosynthesis -> Dry matter | -0.092 | -0.092 | 0.003 | 0 |
| Leaflet age -> Radiation -> Temperature -> Photosynthesis -> Dry matter | 0.542 | 0.542 | 0.002 | 0 |
| Radiation -> Temperature -> Photosynthesis -> Dry matter | -1.923 | -1.922 | 0.005 | 0 |
| Planting strategy -> Gas exchange -> Photosynthesis -> Dry matter | 0.062 | 0.062 | 0 | 0 |
| Leaf adaptation -> Radiation -> Temperature -> Photosynthesis -> Dry matter | -0.017 | -0.017 | 0.001 | 0 |
| Leaflet adaptation -> Radiation -> Temperature -> Dry matter | 0.005 | 0.005 | 0 | 0 |
| Temperature -> Photosynthesis -> Dry matter | -1.912 | -1.912 | 0.005 | 0 |
| Leaflet age -> Radiation -> Temperature -> Dry matter | 0.046 | 0.046 | 0 | 0 |
| Planting strategy -> Radiation -> Gas exchange | 0.374 | 0.374 | 0.001 | 0 |
| Leaf adaptation -> Radiation -> Gas exchange -> Photosynthesis | -0.002 | -0.002 | 0 | 0 |
| Leaf adaptation -> Temperature -> Photosynthesis | 0.005 | 0.005 | 0 | 0 |
| Leaflet age -> Radiation -> Photosynthesis -> Dry matter | -0.78 | -0.78 | 0.002 | 0 |
| Planting strategy -> Photosynthesis -> Dry matter | 0.14 | 0.14 | 0 | 0 |
| Radiation -> Temperature -> Dry matter | -0.164 | -0.164 | 0.001 | 0 |
| Leaflet adaptation -> Radiation -> Temperature -> Photosynthesis -> Dry matter | 0.064 | 0.064 | 0.002 | 0 |
| Leaf adaptation -> Radiation -> Gas exchange | 0.007 | 0.007 | 0 | 0 |
| Leaflet age -> Radiation -> Gas exchange | -0.228 | -0.228 | 0.001 | 0 |
